# 13q mosaic deletion including *RB1* associated to mild phenotype and no cancer outcome – case report and review of the literature

**DOI:** 10.1186/s13039-018-0401-5

**Published:** 2018-09-19

**Authors:** Ilaria Bestetti, Alessandra Sironi, Ilaria Catusi, Milena Mariani, Daniela Giardino, Siranoush Manoukian, Donatella Milani, Lidia Larizza, Chiara Castronovo, Palma Finelli

**Affiliations:** 1 Laboratory of Medical Cytogenetics and Molecular Genetics, IRCCS Istituto Auxologico Italiano, via Ariosto 13, 20145 Milan, Italy; 2 Department of Medical Biotechnology and Translational Medicine, University of Milan, Milan, Italy; 3 Unit of Medical Genetics, Department of Medical Oncology and Hematology, Fondazione IRCCS Istituto Nazionale dei Tumori, Milan, Italy; 4 Medical Genetics Unit, Pediatric Highly Intensive Care, Fondazione IRCCS Ca’ Granda Ospedale Maggiore Policlinico, Milan, Italy

**Keywords:** 13q deletion syndrome, Mosaicism, *RB1*, Array CGH, Interphase FISH

## Abstract

**Background:**

The 13q deletion syndrome is a rare chromosome disorder associated with wide phenotypic spectrum, which is related to size and location of the deleted region and includes intellectual disability, growth retardation, craniofacial dysmorphisms, congenital malformations, and increased risk of retinoblastoma.

**Case presentation:**

Here, we report on a teenage boy with a mild phenotype characterized by obesity, hyperactivity, dysphagia, dysgraphia, sleep disturbance, and minor dysmorphic features (round face, bushy eyebrows, and stubby hands). Array Comparative Genomic Hybridization on blood identified a mosaic 13q14.13-13q31.1 deletion, with a mosaicism rate around 40%, which was confirmed by quantitative PCR and interphase Fluorescent In Situ Hybridization (iFISH) on both blood genomic DNA and cultured/uncultured blood lymphocytes, respectively. Conversely, karyotype analysis on blood estimated a mosaicism rate of 24% and iFISH on buccal smears revealed a borderline value of 0.4%, suggesting the absence of 13q deletion in this cell line.

**Conclusions:**

The comparison with previous patients carrying similar deletions informed that the proband clinical presentation is the mildest reported to date, thus supporting the burden of mosaicism in modulating the phenotype also in case of large chromosomal rearrangements. Characterization of further cases by in-depth mosaicism rate in tissues with different embryonic origins might contribute in the future to a better definition of genotype-phenotype correlation, including tumor risk.

## Background

The 13q deletion syndrome is a rare chromosome disorder characterized by a wide phenotypic spectrum, depending on size and location of the deleted region. Clinical features include moderate to severe intellectual disability (ID), growth retardation, craniofacial dysmorphisms, congenital malformations, and increased risk of tumors, in particular retinoblastoma [1–5]. On the basis of karyotype-phenotype correlation, three patients groups have been distinguished. Group 1 includes patients with proximal deletions (13q12.2q31), who show mild or moderate ID, growth retardation, distal limb anomalies, variable dysmorphic features, and, possibly, retinoblastoma; Group 2 assembles patients with deletions encompassing the 13q32 band and characterized by severe ID and major malformations, mostly affecting the brain; finally, Group 3 comprises patients with distal deletions involving bands 13q33q34, who display severe ID without major malformations [3, 4, 6].

Prenatal diagnosis of 13q deletion syndrome has been scarcely described in literature. Excluding cases with a mosaic r(13) chromosome and those where 13q deletion is the result of an unbalanced reciprocal translocation, up to now seventeen prenatally diagnosed patients have been reported [7, 8], including one mosaic case [9]. While prenatal ultrasound examination is abnormal in almost all the reported patients, with a high frequency of brain anomalies likely due to the almost constant involvement of the q32 band [7, 8], measurement of nuchal translucency (NT) in the first trimester has been rarely recorded [7, 10]. However, as this parameter has been always found increased when evaluated [7, 10], 13q deletion syndrome has been added to the group of those structural chromosomal abnormalities to be considered early in pregnancy (11–14 weeks of gestation) by means of ultrasound assessment [8].

Here, we report on a teenage boy with a mild phenotype who was found to carry a mosaic 13q14.13-13q31.1 deletion, including the retinoblastoma 1 (*RB1*) gene. The comparison with previously described patients clarified that his clinical presentation is the mildest reported to date in association to a 13q deletion, and may be mainly attributed to the mosaic status of the rearrangement. In addition, the in-depth mosaicism rate characterization in tissues with different embryonic origins (blood and buccal epithelium) might contribute in the future to a better definition of genotype-phenotype correlation, including tumor risk. The mosaicism burden in modulating the overall phenotype generally associated to constitutional genetic defects is thus supported also in the case of large chromosomal rearrangements.

## Case presentation

### Clinical report

The patient is a 16-year-old boy, born at term by natural delivery from unrelated healthy parents. The mother and father were 38 and 43 years old at time of birth. Prenatal NT at ultrasound examination and karyotype on amniocytes, performed because of advanced maternal age, were referred as normal. Patient birth weight was 3950 g (90th percentile), length 51 cm (50-75th p), and occipital frontal circumference 34.5 cm (50-75th p). Apgar scores were 9/9. The patient started walking autonomously at 13 months, pronounced his first words at 11 months, and then has shown neither developmental delay (DD) nor ID. Neuropsychological evaluation at 9 years using WISC-III [11] evidenced mild learning disabilities, namely dysgraphia. At the last follow-up at the age of 13, the phenotype was very mild, mainly characterized by obesity (weight > 97th p), a normal height (150.5 cm, 50-75th p), hyperactivity, dysphagia, sleep disturbance, and minor dysmorphic features such as round face, bushy eyebrows, and stubby hands. Brain MRI and angiography showed an altered signal near both the capsular lenticular structures and the head of the caudate nucleus, implying the presence of a hamartoma. He was referred to our lab for Smith-Magenis syndrome (SMS)-like phenotype without SMS molecular diagnosis (neither a 17p11.2 deletion nor *RAI1* mutations were formerly identified).

## Methods

High-resolution array-based Comparative Genomic Hybridization (a-CGH) analysis was performed on genomic blood DNA (gDNA) of the patient and his parents, using the SurePrint G3 Human CGH Microarray Kit 2x400K in accordance with the manufacturer’s instructions (Agilent Technologies, Palo Alto, CA). Copy Number Variants (CNVs) were analyzed and mapped using the Human Genome assembly GRCh37/hg19. The CNV classification by clinical relevance was performed according to the guidelines suggested by Miller et al. in 2010 [12] and successively by the American College of Medical Genetics [13].

To estimate the mosaicism rate based on the a-CGH result, the value of the 13q deletion average log ratio (R_log2_) had been firstly considered. This value is obtained from ADM-2 algorithm (Agilent CytoGenomics v.3.0.6.6, Agilent Technologies) that analyze copy numbers found in patient compared to those found in controls. Using the formula 2^Rlog2^ the unit ratio (R) between patient and controls was calculated. Then, if 2 N and N are the copy number of the target region in the not deleted and deleted 13q cells respectively, and *x* is the percentage of cells carrying the deletion, R can be calculated as follow: $\mathrm{R}=\frac{Patient\ copy\ numbers}{Controls\ copy\ numbers}=\frac{\left(100-x\right)2\mathrm{N}+x\mathrm{N}}{100\left(2\mathrm{N}\right)}$. Namely, in cells without a deletion of the 13q target region ((100 − *x*)2N) and in cells carrying a deletion (*x*N) the percentage of mosaicism for a deletion can be calculated as *x* = 2(1 − R)100.

To confirm a-CGH results, copy number quantification by means of quantitative polymerase chain reaction (qPCR, SYBR® Green methodology) was used. Primers were selected within regions of unique non-repetitive sequence using Primer3 software (http://bioinfo.ut.ee/primer3-0.4.0/); a control amplicon was selected with the same parameters in PCNT at 11q14.1. qPCR conditions and primer sequences are available on request. Standard curve using a dilution series with a control sample mixed with a known non-mosaic deletion of 13q was built up. Conventional cytogenetic analysis was performed on 50 QFQ-banded metaphases obtained from the proband’s peripheral blood lymphocytes using standard procedures. The karyotype was described in accordance with ISCN (2016) [14]. iFISH analysis was performed on either peripheral blood lymphocytes cultivated for 72 h, peripheral uncultured blood lymphocytes, and buccal epithelium using the AneuVysion® multicolor probe LSI 13/21 (Abbott-Vysis, Chicago, IL), that contains both a mixture of unique DNA sequences covering *RB1* at 13q14, and sequences complementary to the D21S259, D21S341, and D21S342 loci within the 21q22.13q22.2 region. In particular, for iFISH analysis on uncultured blood, lymphocytes ring was obtained from Lithium Heparin tubes. The pellet was suspended in a 3:1 methanol:acetic acid fixative solution and dropped across a clean slide. For iFISH analysis on buccal mucosa, the surface cells of both cheeks were first scraped from the patient and two controls by using cytobrushes. The cells were then removed by agitation in a 1:1 methanol:acetic acid fixative solution for 10 min, and the cell suspension was centrifuged at 1200 rpm for 10 min. The pellet was suspended in a 3:1 methanol:acetic acid fixative solution and dropped across a clean slide. The slides were checked with a phase microscope for the presence of cells with adequate morphology. The AneuVysion probe standard protocol was followed with minor modifications. Overall, a total of 500 randomly selected nuclei with both 21q control signals for each tissue were analyzed in both the proband and two control samples. The control with the greatest number of false-positive nuclei was used to determine the normal cutoff. Beta inverse calculation using the Microsoft Excel function, = BETAINV (Confidence level, false-positive cells plus 1, number of cells analyzed) for a 95th confidence limit and a total of 500 observations provided the false positive cutoff values for each tissue [15].

## Results

**Fig. 1:**
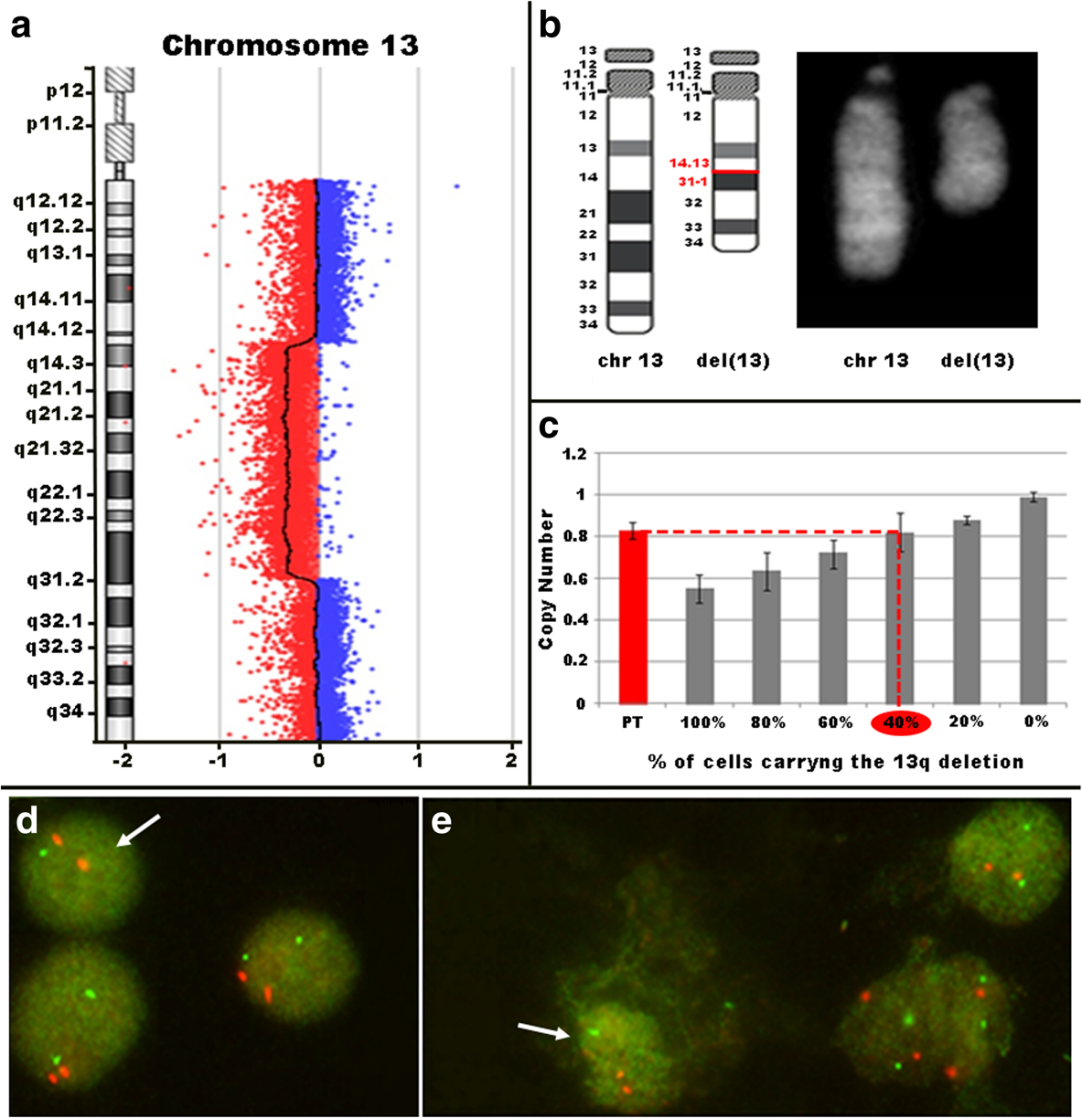
13q mosaic deletion identified in the patient. (**a**) Identification in the patient of a mosaic 40 Mb deletion at 13q14.13q31.1 using Agilent CGH 400 K array. (**b**) Conventional cytogenetic analysis identified the chromosome del(13)(q14.13q31.1) carrying an interstitial deletion in 24% of the analyzed metaphases. (**c**) qPCR estimation (red oval shape) of the deletion mosaicism rate by using both deletion specific probes and a reference mosaic scale including different percentages of cells carrying a 13q deletion. (**d**, **e**) Commercial LSI13/21 probe yielded one hybridization signal specific for the *RB1* locus at 13q14.2 (green) in ~ 35% and 0.4% of patient’s lymphocytes (**d**) and buccal cells (**e**), respectively (arrow), and two signals specific of chromosome 21 (red) in all the analyzed nuclei

a-CGH analysis on patient’s whole blood genomic DNA disclosed a rare ~ 40 Mb 13q14.13q31.1 mosaic deletion (minimum interval, chr13:46,968,080-87,381,985, GRCh37/hg19) as the only pathogenic CNV (http://www.ncbi.nlm.nih.gov/clinvar/, ClinVar accession number SCV000748684), which affected 87 RefSeq genes (Fig. 1a). Somatic mosaicism was estimated at around 40% based on the a-CGH hybridization profile shift and the corresponding log ratio value (− 0.326) (Fig. 1a). The analysis of the parents confirmed the deletion was de novo in origin.

To assess this mosaic deletion by independent techniques, conventional cytogenetic and region-specific genomic analyses were performed. While karyotype analysis pointed out a del(13)(q14.13q31.1) in 24% of the metaphases analyzed (Fig. 1b), qPCR revealed about 40% of deleted cells (Fig. 1c), consistent with the a-CGH results. Similarly, iFISH experiments carried out on patient’s cultured/uncultured blood lymphocytes confirmed the previous a-CGH results. Namely, 13/500 and 10/500 nuclei with only one *RB1* signal were detected in cultured and uncultured controls’ lymphocytes, and the relative false positive cutoff of 4% and 3.3% were applied to patient’s *RB1* deleted nuclei from cultured (195/500) and uncultured (186/500) lymphocytes, leading to a final mosaicism rate of ~ 35% and ~ 34%, respectively (Fig. 1d). A mosaicism underestimation by conventional cytogenetic analysis due to the small size of the sample analyzed was then supported. Indeed, culture-dependent positive selection in favor of dividing cells without the structural aberration has been ruled out by iFISH experiments on both cultured and uncultured lymphocytes that showed the same results.

Contrary, the evaluation of mosaicism using iFISH on buccal smears, which have an embryonic origin different from blood, showed that 4.4% (22/500) of patient’s cells carried the deletion (Fig. 1e). Based on a false-positive cutoff of about 4% of the control nuclei (13/500), the deletion on patient’s buccal smear cells was estimated as 0.4%, a borderline result suggesting the absence of 13q deletion in this cell line.

## Discussion and conclusions

According to the proposed 13q deletion classification [1–6], the deletion identified in the patient falls in Group 1. Although the boy shares with 13q deletion syndrome some neurological features such as sleep disorder, hyperactivity, and learning disabilities, his clinical presentation is definitely the mildest reported to date, as demonstrated by the lack of ID, developmental and growth retardation, peculiar dysmorphisms, major malformations, and tumors (Table 1). This conceptually conflicts with the large size (40 Mb) of the identified structural anomaly as demonstrated by previously reported Group 1 deletions with similar size to that described here, which have been constantly associated to more severe clinical pictures (Table 1) [16–21]. Therefore, the mosaic condition of the deletion might likely explain the patient’s mild phenotype.

**Table 1:** Comparison of phenotypic findings among the reported patients with 13q14q31 deletions and the proband

Reference	Slavotineka and Lacbawana [16]	Caselli et al [17] ^(Case 2)^	Ngo et al [18]	Kogan et al [19]	Malbora et al [20]	Ossandon et al [21] ^(Case 7)^	DECIPHER pt 1732	This report
Cytogenetic bands	13q14.12–q31.2	13q14.11–q31.1	13q14.2–q31	13q14.11–q31.2	13q14.2–q31.3	13q14.11–q31	13q14.11–q31.1	13q14.13–q31.1
Genomic coordinates (GRCh37/hg19)	na	chr13:44,348,617-80,378,610^a^	na	chr13:43,935,154-89,817,398^b^	chr13:47,879,024-90,433,037	na	chr13:44,348,617-80,378,610	chr13:46,968,080-87,381,985 mos
Size (Mb)	na	~ 36	na	~ 46	~ 42.5	~ 38	~ 36	~ 40
Number of RefSeq genes (including *RB1*)^c^	na	105	na	109	85	156	105	87
Inheritance	na	de novo	na	de novo	de novo	na	de novo	de novo
Age (y)	16	2–3	3	< 1, 3	< 2	< 1	3	14
Gender	F	M	F	M	F	na	M	M
Growth retardation	+	+ (<3rd p)	–	+	+	na	–	–
Intellectual disability (moderate to severe)	severe	nr	+	severe	moderate	na	+	–
Developmental delay	+	+	nr	severe	nr	na	–	–
Hypotonia	nr	+	–	+	+	+	–	–
Brain abnormalities	nr	corpus callosum hypoplasia	nr	diffuse polymicrogyria, corpus callosum dysplasia, delayed myelination and prominence of the infra- and supratentorial vasculature	olivopentocerebellar atrophy	corpus callosum dysgenesis	–	cerebral hamartoma
Structural eye abnormalities	diffuse pigmentary retinopathy and atrophy of the optic nerves; bilateral ptosis with strabismus	iris heterochromia	right leukoria	prominent exotropic eyes	ptosis and total ophthalmoplegia at right side, strabismus at left side; bilateral iris heterochromia and telecantus	na	–	–
Retinoblastoma	–	+, unilateral	+, unilateral	+, bilateral	–	+, bilateral	+	–
Gastrointestinal abnormalities	–	–	–	+	–	na	–	–
Limb abnormalities	hands: short, normally positioned thumbs and halluces, brachydactyly of the fingers and toes with nail hypoplasia and fifth finger clinodactyly, second and fourth toes overlapped third toes; feet: bilateral metatarsus adductus and prominent heels	short V toe	–	–	overlapped left toes	na	–	stubby hands
Skeletal abnormalities	scoliosis convex to the right, bilateral contractures of the hips and knees	nr	–	–	left coxa dislocation	na	–	–
Broad forehead	+	+, broad and high	–	+	+	na	+	–
Hypertelorism	+	–	–	–	+	na		–
Broad prominent nasal bridge	+	–, short nose	–	–	+	na	–	–
Malformed ears	large, low-set and posteriorly rotated	+, thick and everted auricular lobes, thick helix	+	–	+, antevert ear lobes	na	+	–
Deeply grooved philtrum	–	+/−	+	–, prominent	+	na	+	–
Downturned mouth	+, wide mouth	–	–	+	–	na	–	–
Thick lower lip	+	+, everted	thin lips	+	–	na	–	–
Cleft palate	+	–	–	+	–	na	–	–
Micrognathia	+	–	–	+	+	na	–	–
Other dysmorphisms	trigonocephaly, sparse hair, upslanted eyebrows, downslanting palpebral fissures, epicanthic folds, anteverted nares, broad nasal tip, irregular dentition with dental crowding, large, one neck pit, a cafe´-au-lait patch on the chest	–	bushy eyebrow, anteverted nostrils, hirsutism	triangular face, downslanting palpebral fissures, bifid uvula	vanished umbilicus	na	–	round face, bushy eyebrows
Other minor anomalies	Tanner stage I female genitalia with opening of the labia majora extended posteriorly to the anus, bilateral inguinal hernias	–	–	bilateral inguinal hernias	–	na	–	–
Medical problems	systolic murmur (grade II/VI) at the left sternal border, right atrial enlargement revealed by echocardiography, hearing loss	minimum aortic reflux	–	hearing loss	lipid abnormality, hypothyroidism	na	–	obesity, hyperactivity, dysphagia, sleep disturbance, dysgraphia

^a^The deletion genomic interval has been deduced from the paper based on the localization of the a-CGH 4x44K kit (Agilent Technologies) oligonucleotide probes

^b^The deletion genomic maximum interval has been deduced from the paper based on the localization of the not deleted SNPs before and after the area of deletion (rs7322455 and rs9522451)

^c^Only coding genes have been considered; each gene has been counted once regardless of the number of its isoforms

–, absent; +, present; *mos* mosaic; *na* not available; *nr* not reported but, possibly, not evaluated

As known, the effect of chromosomal mosaicism on the phenotype cannot be easily predicted as it depends on the different proportions of cells carrying the abnormality which may widely vary in tissues with different embryonic origin, thus supporting the need to precisely assess the mosaicism rate in as many tissues as possible. To date just three cases of a mosaic 13q deletion have been reported [9, 22, 23], although detailed clinical and cytogenetic data are available for just one of them [9]. Widschwendeter and colleagues reported a case of a 25 weeks fetus presenting with multiple anomalies at ultrasound examination, including severe cerebral malformations. Prenatal diagnosis by rapid-FISH on uncultured amniocytes and conventional cytogenetic analysis from cord lymphocyte metaphases revealed a del(13)(q13.3) in 18% of the investigated cells, whose discovery led to the pregnancy termination [9]. Noteworthy, despite the evidence of a quite low mosaic status of the deletion in the tissues analyzed, a very severe phenotype resulted in this case from a rearrangement involving the 13q32 chromosomal segment, whose deletions have been formerly associated to central nervous system anomalies [3, 6]. This evidence confirms how difficult it is to predict the phenotype in case of mosaic genetic defects. This is also true for the present patient where a reverse situation has been observed, with a relatively mild phenotype associated to a significant percentage of blood cells carrying the chromosomal anomaly (~ 40%). By contrast, the observation of a very low-rate mosaicism (0.4%) or the lack of 13q deletion in a tissue with a different embryonic origin, such as the buccal epithelium, allowed us to hypothesize a similar mosaic status likely in all the organs with the same ectodermal origin, such as the brain, which may underlie the patient’s normal neurodevelopment and mild neurological signs [1–6]. Similarly, the absence of 13q deletion or low rate mosaicism might explain the lack of eye tumor, even if retinoblastoma is not a constant feature in association to germline13q deletions involving *RB1* [1–6], and a meta-analysis study showed no difference between children with mosaic and non-mosaic 13q deletion [24]. However the mosaicism has been assessed mostly on blood or fibroblast cells, both of mesenchymal origin [24].

In our opinion, a better definition of genotype-phenotype correlation in case of 13q14 mosaicism, may be achieved evaluating different tissues on multiple patients. It would be ideal to evaluate the mosaic status of a tissue of the same embryonic origin of retina to search for a correlation between the rate of mosaic deletion and retinoblastoma outcome.

Oncologic surveillance may benefit from a precise mosaicism rate assessment on tissues with a different embryonic origin, which is especially relevant in all those mosaic chromosomal conditions involving the deletion of a tumor-suppressor gene, like *RB1* in this case. In our case, the risk to develop tumors of ectodermal origin, may be considered very low, which is consistent with his overcoming childhood without retinoblastoma outcome. An increased risk of sarcoma, which is of mesodermal origin like blood, cannot be completely ruled out. Nevertheless, sarcoma (either soft tissue or bone sarcoma) in hereditary retinoblastoma patients is generally reported as a second tumor subsequent to chemotherapy/radiotherapy [25]. Thus, the patient has been only recommended to avoid unnecessary radiation exposure and, in the absence of specific symptoms, undergo a periodical physical examination. Interestingly, very low-level ectodermal mosaicism may also imply a small percentage of germinal cells carrying the deletion, thus resulting in a low reproductive risk. In addition, it may also explain why the deletion here described was not prenatally detected during amniocentesis, as amniotic cells, which are assumed to closely reflect the karyotype of the fetus, are most of ectodermal origin [26].

In conclusion, the patient reported here confirms that the mosaic status, also of large chromosomal rearrangements, can considerably modulate the phenotype associated to constitutional genetic defects. The assessment of mosaicism rate in more than one tissue of different embryonic origin may enhance genotype-phenotype correlation. In addition, the present case underlies that amniocentesis misdiagnosis is a concrete eventuality in patients bearing low level fetal chromosomal mosaicism, which cannot be easily identifiable in the absence of ultrasound indications.

## Abbreviation

a-CGH: Array Comparative Genomic Hybridization
CNVs: Copy Number Variants
DD: Developmental delay
gDNA: Genomic DNA
ID: Intellectual disability
iFISH: Interphase Fluorescence in situ hybridization
MRI: Magnetic Resonance Imaging
NT: Nuchal translucency
qPCR: Quantitative PCR
R: Unit ratio
R_log2_: Average log ration
SMS: Smith-Magenis Syndrome
WISC: Wechsler Intelligence Scale for Children
